# Clinical Remarks by Prof. W. Parker—Fissures of the Rectum

**Published:** 1849-07

**Authors:** 


*Clinical Remarks by Prof. W. Parker—Fissures of the Rectum.*—A the same Clinique a man presented himself with some exceedingly painful affection of the Rectum, which he termed hemorrhoids, or piles, and for which he had been under treatment a considerable time. Dr. Parker observed that in all such affections of the rectum, it was of the utmost importance to make an early and efficient examination. Pain in that region, especially on going to stool, may arise from a variety of morbid conditions, such as hemorrhoids, fissures, strictures, and neuralgia; each of which requires its own peculiar treatment.

And yet, except in these cases of hemorrhoids where the tumors project externally, these affections cannot be distinguished with actual certainty, without introducing the finger into the rectum and making a careful manual examination. In the case before us, there has been long continued and obstinate costiveness, with consequent impairment of digestion; and every attempt at stool causes an exceedingly severe stinging, smarting pain, amounting often almost to perfect agony, and it continues with greater or less intensity for several hours after the evacuation. The stools are often streaked with blood, and at times the hemorrhage is considerable. On introducing the finger into the rectum, no hemorrhoidal tumors or stricture can be discovered; but by carefully pressing the finger in one direction and then in another, until you have brought it in contact successively with the whole inner surface of the part, you find no difficulty until you press directly against the posterior surface, when great pain is immediately induced; and with a little care the fissure or crack in the mucous membrane can be distinctly felt. In making such examinations care must be taken to get the distinct attention of the patient to the pressure in each successive direction, because great complaint is frequently made on first introducing the finger without referring it to any particular spot. But if the patient be requested to exercise patience, and the surgeon proceeds with care, he will soon find the point affected. Having ascertained the existence and location of the fissure, the next inquiry is in reference to a proper remedy. This, said Dr. Parker, consists in dividing the sphincter ani freely, so as to allow the bowels to be evacuated without putting the rectum on the stretch at all. And while the divided sphincter is re-uniting, the fissure heals and the patient is cured. There is no other reliable mode of cure, for so long as the sphincter is allowed to remain intact, the effort necessary to overcome it in evacuating the bowels, almost necessarily puts the mucous membrane so much on the stretch as to effectually prevent a closure of the fissure.

The object in dividing the sphincter, being to allow the mucous membrane to remain at rest or unstretched long enough to heal firmly, it matters very little whether the incision through it corresponds with the location of the fissure or not. Sometimes internal hemorrhoids and fissures both exist in the same subject, and the practitioner cannot be too careful in examining, distinguishing, and properly treating these painful affections.—*Annalist.*

